# Occupational differences in COVID-19 incidence, severity, and mortality in the United Kingdom: Available data and framework for analyses

**DOI:** 10.12688/wellcomeopenres.16729.2

**Published:** 2023-01-13

**Authors:** Neil Pearce, Sarah Rhodes, Katie Stocking, Lucy Pembrey, Karin van Veldhoven, Elizabeth B. Brickley, Steve Robertson, Donna Davoren, Vahe Nafilyan, Ben Windsor-Shellard, Tony Fletcher, Martie van Tongeren

**Affiliations:** 1 Faculty of Epidemiology and Population Health, London School of Hygiene & Tropical Medicine, London, WC1E 7HT, UK; 2 University of Manchester, Manchester, M13 9PL, UK; 3 Faculty of Public Health Policy, London School of Hygiene & Tropical Medicine, London, WC1E 7HT, UK; 4 Office of National Statistics (ONS), London, SWIV 2QQ, UK

**Keywords:** COVID-19, occupation, epidemiology

## Abstract

There are important differences in the risk of SARS-CoV-2 infection and death depending on occupation. Infections in healthcare workers have received the most attention, and there are clearly increased risks for intensive care unit workers who are caring for COVID-19 patients. However, a number of other occupations may also be at an increased risk, particularly those which involve social care or contact with the public.

A large number of data sets are available with the potential to assess occupational risks of COVID-19 incidence, severity, or mortality. We are reviewing these data sets as part of the Partnership for Research in Occupational, Transport, Environmental COVID Transmission (PROTECT) initiative, which is part of the National COVID-19 Core Studies. In this report, we review the data sets available (including the key variables on occupation and potential confounders) for examining occupational differences in SARS-CoV-2 infection and COVID-19 incidence, severity and mortality. We also discuss the possible types of analyses of these data sets and the definitions of (occupational) exposure and outcomes.

We conclude that none of these data sets are ideal, and all have various strengths and weaknesses. For example, mortality data suffer from problems of coding of COVID-19 deaths, and the deaths (in England and Wales) that have been referred to the coroner are unavailable. On the other hand, testing data is heavily biased in some periods (particularly the first wave) because some occupations (e.g. healthcare workers) were tested more often than the general population. Random population surveys are, in principle, ideal for estimating population prevalence and incidence, but are also affected by non-response. Thus, any analysis of the risks in a particular occupation or sector (e.g. transport), will require a careful analysis and triangulation of findings across the various available data sets.

## Introduction

Since March 2020, there have been epidemics of SARS-CoV-2 infection throughout most parts of the world ^1, 2^, and the United Kingdom has experienced particularly high infection and death rates. There are major occupational differences in the risk of SARS-CoV-2 infection and death ^3– 5^, but there have been relatively few systematic analyses of infection or death rates across different occupation types. There are clearly increased risks for intensive care unit workers who are caring for COVID-19 patients, as well as increased risks for other health and social care workers. However, a number of other occupations may also be at an increased risk, particularly those which involve social care or contact with the public ^5^.

A large number of data sets are available to potentially assess occupational risks of COVID-19 incidence, severity, or mortality ( Table 1) in the United Kingdom (UK). We are reviewing these data sets as part of the Partnership for Research in Occupational, Transport, Environmental COVID Transmission ( PROTECT) initiative, part of the National COVID-19 Core Studies. In this report, we review the available data sets, and in the Discussion, we provide more detail on some of the larger and more relevant data sets available for examining occupational differences in SARS-CoV-2 infection and COVID-19 incidence, severity and mortality. We also discuss the possible types of analyses of these data sets and the definitions of (occupational) exposure and outcomes.

**Table 1.** Possible data sets for occupational analyses.

Data source	Occupation data	Confounders	Outcome data	Availability to researchers?
**Office of National Statistics (ONS) data**				
Numerator: ONS registered deaths Denominator: Annual Population Survey	Current occupation	Age, sex, residence, deprivation, ethnicity	COVID-19 on death certificate	Yes
**National Health Service (NHS) data**				
NHS hospitalization data	No	Age, sex, residence, deprivation, ethnicity	COVID-19 admission	Yes
NHS workplace absence reports	Current occupation	Unknown	COVID-19 as cause	Yes
OpenSafely	No	Age, sex, residence, deprivation, ethnicity, comorbidities	Hospital death from COVID-19	Yes
**HSE data**				
Workplace sickness reports (RIDDOR)	Current occupation	No	COVID-19 as cause	Yes
**Population cohorts**				
UK Biobank + many others	Most are using Wellcome COVID-19 questionnaire; includes employment, sector, travel to work	Multiple	SARS-CoV-2 positive test	Yes (UK Biobank)
**Population surveys**				
ONS Infection Survey	Current occupation, working from home, transport	Age, sex, ethnicity, geographical identifiers	Series of SARS-CoV-2 tests	Yes, under application
Understanding Society	Employment, work conditions, travel to work - key work sector, industry, 3-digit SOC code.	Age, sex, ethnicity, geographical identifiers	SARS-CoV-2 test Symptoms	Yes
Real-time Assessment of Community Transmission (REACT)	Unknown	Yes	COVID-19 symptoms and test results	Possible
**Apps**				
ZOE (King’s College)	Not currently	Yes	COVID-19 symptoms and test results	Possible
**Population testing**				
Pillar 1	No	No	SARS-CoV-2 test	Possible
Pillar 2	No	No	SARS-CoV-2 test	Possible
Test-Trace-Isolate (TTI)	No	No	Close contacts	Possible
**HSE/PHE data**				
Publica Health England (PHE) outbreak investigations ^10^	Current occupation and exposures	Age, sex, residence, deprivation, ethnicity	SARS-CoV-2 test	Yes

## Study designs

### Source population and study population

In any analyses of this type, one may distinguish several populations that are relevant:

- There is a target population to which we wish to draw inferences (e.g. all people in the UK, all people on the planet)
- There is a source population which is used as the source of participants for a particular study (e.g. everyone living in the UK aged 20–64 and in employment)
- There is a (perhaps smaller) study population (i.e. the group of people who actually take part in the study, with some of the source population not taking part either due to selection by the investigators, or self-selection (i.e. non-response))

Since the focus is on occupational exposure to COVID-19, the focus of almost all analyses will be on the working age population and will usually be restricted to those who were in employment at the beginning of the pandemic on 11 March 2020 ^6^. In data sets such as the Office of National Statistics (ONS) mortality data, the source population is the entire population of England and Wales (aged 20–64 and in employment at the beginning of the pandemic, and with an occupation recorded). In other data sets, e.g. UK Biobank, the source population is the entire population of England and Wales, aged 40–69 years and living in the UK in 2006, and who have not emigrated subsequently; the study population is those who actually took part in the survey (response rate = 5.5%).

### Cohort data

Cohort data includes national mortality data (ONS data), cohorts based on Electronic Health Records (EHRs) such as OpenSafely, as well as population cohorts such as UK Biobank and many others (this data is being integrated and standardised, to the extent possible, by the Longitudinal Health Core Study, and the Data and Connectivity Core Study ( National COVID-19 Core Studies)). Most cohorts have, or will have, linked mortality data. Many also have SARS-CoV-2 testing data, either as a single test, as a series of repeated test results, or self-reported tests and symptoms. Some also have hospitalization data.

### Case-control data

In some instances, case-control studies can be nested within cohorts, or can be conducted as ‘stand-alone’ studies. One particular instance of this is the test-negative design ^7, 8^. It has been proposed that this is used for COVID-19 research for populations in which not everyone has been tested. The logic is that there are many individual factors (health seeking behaviour, access to transport, etc.) which may influence someone’s ability to get tested. Thus, if we compare those who test positive with general population control samples, there may be considerable bias. When the test-negative design ^7, 8^ is applied to COVID-19, people who are tested are given the questionnaire on risk factors (or we obtain risk factor information some other way), and we then compare those who test positive with those who test negative. If everyone in the study population is tested (i.e. a comprehensive investigation), then this is essentially a cross-sectional study. However, in cases where not everyone is tested, then we compare the test-positives with the test-negatives. It should be noted that people may be tested because they have symptoms, and therefore those who test negative may have a different respiratory infection. Thus, when we compare these two groups, we can learn about risk factors that are specific for SARS-CoV-2 (rather than respiratory infections in general). We can learn even more if we can also give a questionnaire to an additional carefully selected control person who was not symptomatic and therefore not tested. By comparing the test-positives with their controls, we can learn about risk factors for SARS-CoV-2, and by comparing the test-negatives with their controls, we can learn about risk factors for other respiratory infections. By putting the three sets of analyses together ^7^, i.e. test+ves vs test-ves, test+ves vs additional selected controls, test-ves vs population controls – using triangulation ^9^ – we can learn a great deal.

### Cross-sectional data

Cross-sectional surveys include the baseline surveys for cohort studies (e.g. if everyone has a SARS-CoV-2 test at baseline), and ‘one-off’ outbreak investigations. Essentially, if everyone is only tested once, then usually the study will be cross-sectional. Such surveys can be analysed in the same way as a case-control study ^11^.

## Outcome variables

The outcome data will vary according to the data set under analysis. It can include measures of SARS-CoV-2 infection (symptoms, positive test results), severity (hospitalisation, intensive care unit (ICU) admission) or mortality (COVID-19-related death, excess mortality). In most analyses one would take the first positive test result by reverse transcription polymerase chain reaction (RT-PCR) or serology as an outcome. One would only consider multiple positive test results in the same person if it were considered that these involved different infections. Testing data is particularly difficult to interpret, because difference occupations are likely to be tested with different frequencies, and for different reasons (i.e. routine testing, symptomatic testing, testing of close contacts).

There are a number of different classification methods for symptoms ^12^, for example, the ‘any symptom that could be caused by Coronavirus’ definition applied by Understanding Society ^13^. Other methods include focussing on three key symptoms ^14^ or applying a prediction model ^15^.

There are also a number of ways to classify death from COVID-19 ^16^, for example, some methods include those where COVID-19 is mentioned on the death certificate ^17^, whereas others classify them as, ‘any death within 28 days of a positive test’, as seen on the GOV.UK website.

## Exposure variables

The analyses described in this document focus on the relationship between occupation and work-related risk factors and health outcomes. An ideal investigation into the risk of transmission and infection in the workplace would include data that indicates the (likelihood of) exposure to infected people. However, this is virtually impossible, perhaps with the exception of healthcare staff working in COVID-19 wards. Hence, markers for the risk of exposure in groups of workers (rather than individuals) will need to be developed. In occupational epidemiological studies, different methodologies have been used to assess exposures to hazardous agents (or markers of exposure) in workplaces. Ideally, exposure is assessed quantitatively based on measurements of the environments. This is extremely challenging for SARS-CoV-2 due to the transient nature of the exposure. One possible option for future research may be to measure SARS-CoV-2 in sewage waste from workplaces, in order to determine if infections are occurring, and some trials are ongoing ^18^. However, such data are unlikely to be widely available, and it will not be possible to use such data to distinguish between the exposure of individual workers within the same workplace.

### Occupational questionnaires

Information on occupational risk factors can be collected through questionnaires. Many of the studies and data sources reported in Table 1, will include data from questionnaires completed by participants. Unfortunately, the level and detail of occupational information requested in the questionnaires varies widely between the different data sources and studies. Some will have very limited data, e.g. just whether participants are working from home or are furloughed, working hours (e.g. full-time or part-time work), patterns (shift-work), or job security (e.g. zero hours contracts). Further details can be collected by questionnaires, and an example of a questionnaire which aims to collect data on work-related risk factors is described in *Extended data* ^19^.

### Occupational codes

Analyses of health outcomes, including symptoms, positive tests, hospitalisation, ICU admissions, and deaths for each occupational group is informative. Ideally, occupational data should be collected and analysed using standard occupational classification (SOC), such as SOC2010 or SOC2020. The SOC codes are specific to the UK, but are closely related to the International Standard Classification of Occupation (ISCO) codes. The use of the SOC will allow better comparison across studies. In this classification, each occupation is given a 4-digit code, but analyses can also be done using just the first digit, first two digits, etc. (see Discussion for 1- and 2-digit SOC codes). Analyses using 4-digit codes may not always be possible due to the size of the study, however, when possible, they may provide very useful information. For example, the first ONS report on COVID-19 deaths and occupation ^20^ demonstrated that within the broad category of Road Transport Driver (SOC 821), the COVID-19 mortality rate was elevated in bus and taxi drivers, but not in large goods vehicle and van drivers, suggesting that contact with the general public is a risk factor.

3-digit and 4-digit occupational codes can be selected and grouped on the basis of prior knowledge. One example of this is given in the first ONS report which covers the first few months of the pandemic in the UK ^20^ (see Table 2).

**Table 2.** Age-standardised mortality per 100,000 in selected Standard Occupational Classification (SOC) unit groups.

SOC Unit Group	Occupation	Mortality Rate per 100,000	Lower CI	Upper CI
8211	Large goods vehicle drivers	9.8	6.5	14.1
8212	Van drivers	12.6	8.5	18.0
8213	Bus and coach drivers	26.4	17.1	38.4
8214	Taxi and cab drivers and chauffeurs	36.4	28.6	45.6
	All men aged 20 to 64 years	9.9	9.4	10.4

Similar analyses have been done grouping healthcare workers and social care workers ^17^. Some analyses have also been reported by industry sector, and it has been possible to group occupations and sectors into “essential” and “non-essential” workers, i.e. those who were required to go to work throughout the pandemic, and those who were able to work from home ^21^.

### Occupational Self-Coding and Automatic Recording (OSCAR)

One barrier for using SOC or other standardised occupational classifications is that they generally require collection of information on job and activities using free text questions, combined with post-hoc coding. This can be very time consuming, although some tools are available that can be used for (semi-)automatic coding e.g. Computer Assisted Structured Coding Tool ( CASCOT). Still, many researchers are not keen to include open-ended and free text questions. 

To overcome this problem, an occupational self-coding tool was developed for a study on chronic obstructive pulmonary disease (COPD) using the UK Biobank ^20^. Occupational Self-Coding and Automatic Recoding ( OSCAR) was developed by the authors using the hierarchical structure of the SOC2000 which allows individuals to collect and automatically code their lifetime job histories via a simple decision-tree model ^20^. 

We are currently modifying OSCAR in order to focus only on recent occupations (e.g. since the beginning of 2020, rather than a full history). In addition, we have developed a more detailed occupational questionnaire as an optional tool in the COVID-19 version of OSCAR (see *Extended data* ^19^).

### The COVID-19 Job-Exposure-Matrix (JEM)

The SOC codes can also be used in combination with a Job Exposure Matrix (JEM) which has now been developed and published ^22^. This approach has been used successfully in many other occupational epidemiological studies based on general population data ^23^, where limited data are available on work-related factors. A JEM is basically a table that provides an estimate of exposure for each occupation. Further extensions can be made by including time period or other factors. The exposure estimate can be a dichotomous variable (YES vs NO), an ordinal scale (e.g. low, medium, and high) or a quantitative estimate (e.g. concentration in air). The COVID-19 JEM is being developed in collaboration with researchers in the United Kingdom, The Netherlands, and Denmark. The JEM enables the assessment of risk factors for all 4-digit occupational codes. Risk factors for transmission, included in the JEM are:

a. Number of workers at worksite during a typical workday
b. Nature of contacts with co-workers, general public, or patients with COVID-19
c. The risk through contaminated work surfaces and materials
d. Location of work: indoors or outdoors
e. The possibility to keep at least 1m of social distance
f. The need and usage of face covering
g. Job insecurity
h. Proportion of migrant workers

For each of these dimensions the jobs are classified into:

- No risk (0)
- Low risk (1)
- Elevated risk (2)
- High risk (3)

Occupations are classified for each of these factors as follows:

-   0 (0)

-   1–10% (1)

-   11–25% (2)

-   >25% (3)

The JEM is developed based on a combination of data and expert judgement which are used to classify each occupation, e.g. according to the likelihood/extent of public contact. As the JEM is developed in collaboration with European partners, an international occupational classification system (ISCO) is used, rather than the UK SOC classification, but it has now been translated into SOC, and used for analyses of SARS-CoV-2 infection survey data ^24^. These found that the first six domains, but not the last two, were associated with increased risk of infection, particularly during the first wave of the pandemic.

## Confounders and effect modifiers

When considering differences in SARS-CoV-2 and COVID-19 risk in different occupations, the ‘standard’ confounders include age, sex, ethnicity, deprivation, and region. Some of these factors may be time-varying, and this should ideally be taken into account in the analysis.

### Race/ethnicity

The term ‘race’ is an artificial construct, and therefore most researchers prefer to use the term ‘ethnicity’ ^25^ which is a complex construct that includes biology, history, cultural orientation and practice, language, religion, and lifestyle, all of which can affect health. The UK census reports 18 categories of ethnicity ( Table 3). Although it may be necessary to group these 18 categories into two – White and BAME (Black Asian and Minority Ethnic) - when study numbers are small, many object to this categorisation on the basis that there are substantial differences (including experiences of racism as well as cultural, social, economic, historical factors) between the different ‘non-White’ ethnic groups; thus it is preferable to report study findings separately for each ethnic group if the numbers permit. For example, one recent analysis ^26^ of COVID-19 infection, hospitalisation, and mortality reported the findings by separating ethnicities into White (63%), South Asian (6%), Black (2%), Other (2%) and Mixed (1%) with 26% not providing any information on ethnicity. It should be acknowledged that analyses of Covid-19 by ethnicity are complex, since there are likely to be differences in risk of infection, comorbidities, probability of being tested, and quality of health care. For example, Hawkins *et al.* ^28^ found that Blacks consistently had higher mortality rates from Covid-19 than Whites within the same occupation.

**Table 3.** Ethnicity information available from the UK census ^27^.

Code	Name
01	White: English/Welsh/Scottish/Northern Irish/British
02	White: Irish
03	White: Gypsy or Irish Traveller
04	White: Other White
05	Mixed/multiple ethnic groups: White and Black Caribbean
06	Mixed/multiple ethnic groups: White and Black African
07	Mixed/multiple ethnic groups: White and Asian
08	Mixed/multiple ethnic groups: Other Mixed
09	Asian/Asian British: Indian
10	Asian/Asian British: Pakistani
11	Asian/Asian British: Bangladeshi
12	Asian/Asian British: Chinese
13	Asian/Asian British: Other Asian
14	Black/African/Caribbean/Black British: African
15	Black/African/Caribbean/Black British: Caribbean
16	Black/African/Caribbean/Black British: Other Black
17	Other ethnic group: Arab
18	Other ethnic group: Any other ethnic group
XX	No code required

### Region

The UK census has 10 categories for regions in England and Wales ( Table 4). Each region (with the exception of London) includes a mix of urban and rural residents. More detailed information is also available, down to postcode level, which enables comparisons of Covid-19 risks between urban and rural areas, and adjustment for population density and urban/rural status ^21^.

**Table 4.** Regional information available from the UK census ^27^.

Code	Name
E12000001	North East
E12000002	North West
E12000003	Yorkshire and the Humber
E12000004	East Midlands
E12000005	West Midlands
E12000006	East of England
E12000007	London
E12000008	South East
E12000009	South West
W92000004	Wales

### Deprivation

The UK census has five categories of household deprivation ( Table 5).

**Table 5.** Household deprivation categories in the UK census ^27^.

Code	Name
1	Household is not deprived in any dimension
2	Household is deprived in 1 dimension
3	Household is deprived in 2 dimensions
4	Household is deprived in 3 dimensions
5	Household is deprived in 4 dimensions
X	No code required

There are also several potential effect modifiers, including working from home, being furloughed, and the availability and use of personal protective equipment (PPE). All of these may modify the risk of infection, even if remaining in the same job throughout the pandemic.

## Statistical analyses

### Descriptive analyses

All analyses will usually start with similar descriptive analyses, e.g. tables of the characteristics of the study participants. Intersectoral approaches may also be used in these descriptive analyses. These will usually be specific to the data set under analysis, so we will not try to establish general principles here.

### Directly age-standardised rates

The main studies that have used directly age-standardised rates are the ONS analyses ^20^. These have estimated age-standardised mortality rates (ASMR) standardised to the 2013 European Standard Population. They are described in more detail in the Discussion section.

### Poisson regression (or Cox proportional hazard analyses)

Cohort studies that have more comprehensive data, including data on potential confounders, can be analysed using Poisson regression ^29^ or the Cox proportional hazards model (they should yield the same results). For each occupational group being considered (see below for how these are defined and compared), we might run the following models if we are specifically investigating occupational exposures, and we wish to adjust for confounders such as ethnicity, deprivation, etc:

Outcome                    Independent variables
  COVID-19 death       occupation, age, sex
  COVID-19 death       occupation, age, sex, ethnicity
  COVID-19 death       occupation, age, sex, deprivation
  COVID-19 death       occupation, age, sex, region
  COVID-19 death       occupation, age, sex, ethnicity, deprivation, region

The main aims are to:

- Ascertain which occupations are at greatest risk of COVID-19 and/or death from COVID-19
- Ascertain to what extent these increased risks are explained by confounding by ethnicity, deprivation, or region (this requires a specific causal model because, for example, ethnicity can affect the likelihood of deprivation, etc.)

### Excess mortality analyses

There is a considerable amount of literature on the use of excess mortality analyses for studying COVID-19 mortality ^30^. The rationale is that excess all-cause mortality may, in some instances, be a better measure of the true mortality burden from COVID-19 than is the case for COVID-19-specific mortality, because of the problems of classification of COVID-19 death on death certificates ^1, 2^. For example, Vandoros ^31^ used ONS data on the number of deaths in England and Wales that did not officially involve COVID-19 over the period 2015–2020; they used a difference-in-differences econometric approach to study whether there was a relative increase in deaths not registered as COVID-19-related during the pandemic, compared to a control time period. Results suggest that there were an additional 968 weekly deaths that officially did not involve COVID-19, compared to what would otherwise have been expected. Vandoros concluded that it is possible that some people are dying from COVID-19 without being diagnosed, and/or that there are excess deaths due to other causes resulting from the pandemic.

### Logistic regression

Case-control studies can be analysed using logistic regression ^29^. The general modelling strategy is essentially the same as that described for Poisson regression or the Cox proportional hazards model (see above).

### Triangulation of analyses

The idea of ‘triangulating’ evidence from different methods and data sources has been proposed and used implicitly for decades, often without explicitly describing it as triangulation ^9, 32, 33^. The key aspect of triangulation is that it involves comparing results from at least two (but ideally more) methods that have differing key sources of unrelated bias ^9^. If evidence from such different epidemiological approaches all point to the same conclusion, this strengthens confidence that that is the correct causal conclusion, particularly when the key sources of bias for some of the approaches predict that the findings would point in opposite directions. The difference between ‘epidemiologic triangulation’ and the systematic review or meta-analysis of trials or epidemiological studies is that a systematic review seeks similar studies, which are expected to yield similar findings, and hence can be grouped in a meta-analysis to obtain a more precise estimate of an exposure. Epidemiological triangulation, in contrast, looks for different types of studies, which might be expected to yield different findings, because they involve different potential biases, or biases in different directions; this allows one to assess the likely existence or absence of the biases that one might be concerned about in one particular type of study ^34^. Triangulation is particularly relevant to analyses of the relationship between COVID-19 and occupation, since the available databases have different strengths and weaknesses, often with biases in different directions. Thus, it is important to compare findings for a particular occupation (e.g. healthcare workers) across different data sets, and to attempt to understand why different analyses may give different results, and what the potential strengths and directions of the biases are in the different data sets. For example, analyses early in the pandemic reported very high relative risks for infection in health care workers, in contrast with only moderately elevated risk of mortality for the same occupations ^21^. This is likely to be due to the frequent testing of health care workers (much more frequently than the general population) during the first wave of the pandemic ^35^.

### Meta-analysis

Meta-analysis ^36^ is a quantitative technique that allows the combination effect measures from multiple studies to increase precision and to allow for an overall summary. Meta-analysis is often accompanied with forest plots ^37^, which allow visual comparison of effect measures, to assess consistency and explore variation.

An advantage of analysing multiple data sets using the same general protocol is that there will be consistency in terms of the chosen outcome measures, the summary measures used, the format of the occupation variables, and the confounders adjusted for. However, in this context meta-analysis must be approached very cautiously because of the complex heterogeneity among the data sets in terms of the methods of data-collection, outcome measures, time periods covered, and testing strategies.

Occupations can be grouped in many different ways and the comparison of multiple occupation groups will lead to a large number of effect measures that are likely to be unsuitable for meta-analysis. The use of the JEM (see below) will allow us to look at the effect of a small number of key exposure variables related to occupation. Meta-analysis could then be performed on the effect measures related to these exposures.

### Analysis strategy

There is a variety of analysis strategies which are used in analyses of this type, and there is no single ‘gold standard’ that can be universally applied ^38, 39^. One possible analysis strategy would involve considering the following contrasts:

- 1-digit occupational groups (either all other occupations, or SOC Group 1, used as the reference)
- 2-digit occupational analyses (either all other occupations, or SOC Group 1, used as the reference)
- Selected 3-digit and 4-digit occupational groups (either all other occupations, or the relevant 1-digit SOC Group used as the reference)
- JEM (as a continuous or categorical exposure variable)

For each occupational group being considered, when the relevant variables are available, we would run the following Poisson regression models:

Outcome                    Independent variables
  COVID-19 death       occupation, age, sex
  COVID-19 death       occupation, age, sex, ethnicity
  COVID-19 death       occupation, age, sex, deprivation
  COVID-19 death       occupation, age, sex, region
  COVID-19 death       occupation, age, sex, ethnicity, deprivation, region

The main aims are to:

- Ascertain which occupations are at greatest risk of COVID-19 and/or death from COVID-19
- Where possible, ascertain to what extent these increased risks are explained by confounding by ethnicity, deprivation, or region

## Discussion

In this section we discuss the key data sets associated with this study in further detail.

### ONS mortality data

***Study type: cohort***

*Possible analyses: age-standardized rates, Poisson regression, Cox proportional hazards model*

The Office for National Statistics (ONS) has recently published several reports on COVID-19 deaths in the working age population (20–64 years) in England and Wales ^20^. There were high COVID-19 death rates in selected occupations, particularly for men, including high death rates in occupations involving public contact ^40, 41^. These job types include security guards, taxi drivers and chauffeurs, bus and coach drivers, chefs, sales and retail assistants, and social care workers.

The findings were adjusted for age, but not for other factors such as ethnic group, place of residence and deprivation. In the ONS data, deaths were defined using the International Classification of Diseases, 10th Revision (ICD-10). Deaths involving the coronavirus (COVID-19) include those with an underlying cause, or any mention of ICD-10 codes U07.1 (COVID-19, virus identified) or U07.2 (COVID-19, virus not identified). ONS applied an age restriction, selecting deaths among those aged 20 to 64 years, because of limitations of occupational mortality data for those below the age of 20 years and those above the age of 64 years. Occupation is reported on the death certificate at the time of death registration by the informant. This information was then coded using SOC2010.

Population counts for occupations were obtained from the Annual Population Survey (APS), using data collected in 2019 ^17, 42^. The APS is the largest ongoing household survey in the UK and is based on interviews with members of randomly selected households. The survey covers a range of diverse topics, including information on occupation, which is then coded using the SOC2010 Manual ^43^. The population counts were also restricted to those aged 20 to 64 years and were weighted to be representative of those living in England and Wales.

Mortality rates for the broader population of all usual residents in England and Wales were based on the mid-year population estimates for 2018.

*Unlinked data*

This is the ‘standard’ way of conducting such analyses, which has been used in the ONS reports to date, where the numerator data is obtained from death registrations, and the denominator data is obtained from population surveys. The relevant files are death registrations, England and Wales and the Annual Population Survey (see Table 1).

*Linked data*

This is a data set newly available from ONS ^44^. The 2011 census was linked to the 2011–2013 Patient Registers (PR) using deterministic and probabilistic matching. It was first linked deterministically using 24 different matching keys, based on a combination of forename, surname, date of birth, sex, and geography (postcode or Unique Property Reference Number). Using different combinations of these variables ensured that records that contain errors in these variables could nonetheless be linked. The matches needed to be unique within a matching key for the match to be accepted. Probabilistic matching was then used to attempt to match records that were not linked deterministically, using 13 different combinations of personal identifiers. Candidate matches were assigned to census records using the Felligi-Sunter probabilistic matching method. 

Of the 53,483,502 census records, 50,019,451 were linked deterministically. A total of 555,291 additional matches were obtained using probabilistic matching. This linkage enabled the NHS number to be added to the census 2011 records in order to facilitate the linkage to the death registration data.

Deaths were linked to the 2011 census using NHS Number, and 89.9% of deaths that occurred between 27 ^th^ March 2011 and 1 ^st^ March 2020 were linked to the 2011 census. Initially, ONS-linked deaths occurring between 2 ^nd ^March 2020 and 14 ^th^ July 2020 that were registered by 28 ^th^ July 2020, were linked to the census file using NHS Number and a deterministic match key linkage method where NHS Number was unavailable, achieving a linkage rate of 90.2% of deaths. The unmatched deaths comprise people not present in the UK at the 2011 census, people who arrived in the UK in the year before the census (and were excluded from the study), and people who were present at census but not enumerated in the census.

The study dataset does not contain any information on whether individuals have left the country. To avoid biasing the denominators, ONS derived and applied weights reflecting the probability of having remained in the country between March 2011 and March 2020, based on data from the NHS Patient Register and the International Passenger Survey (IPS).

Despite being in the population at risk of COVID-19-related death in March 2020, ONS did not replenish the sample with post-2011 births or immigrants. While the latter group could have been identified and in principle linked to our data, neither group are captured in the 2011 census and therefore they have no ethnicity or covariate data recorded. Additionally, the younger population have been the least affected with COVID-19 related hospitalisation and mortality. For the same reason, individuals not enumerated at the 2011 census (estimated to be 6.1% of the population of England and Wales) were not included in the study population.

At this stage, the data set only includes deaths for 2020, but it is possible that deaths from 2011–2019 could also be linked.

### UK Biobank

***Study type: cohort, nested case-control (test-negative design)***

*Possible analyses: Poisson regression, Cox proportional hazards model, logistic regression*

UK Biobank is a population-based prospective study involving 502,506 participants throughout England and Wales ^35^, recruited during 2006–2010. The study had a very low response rate (5.5%) ^45^, meaning that the initial cross-sectional baseline analyses are likely to be subject to selection bias, but this is less likely to affect analyses based on subsequent follow-up over time ^46^. At the latest follow-up (pre-pandemic), 14,423 participants had died, leaving 488,083 living participants around the time that the COVID-19 pandemic commenced.

UK Biobank has baseline information on a large number of variables, including demographic and social data, health risk data, medical factors, and environmental exposures. The demographic variables include age, sex, and ethnicity (defined as White, Black, and Other). Social variables include education, housing, and household income. Current occupation was recorded at recruitment (during 2006–2010), and this has been coded using SOC2000 codes ^47^.

UK Biobank also includes the results of COVID-19 tests from Public Health England’s Second Generation Surveillance System microbiology database ^35^. Chadeau-Hyam *et al.* ^35^ recently analysed this data which included the results from 7,539 tests from 4,509 UK Biobank participants between 16 ^th^ March and 18 ^th^ May 2020. More recently, Mutambudzi *et al.* ^47^ analysed the COVID-19 test results for the period 16 ^th^ March to 26 ^th^ July 2020 in relation to occupation. They found that there were 120,075 working participants aged 49–64 years in 2020, after excluding those who had died previously, or had missing data. They compared the occupation at baseline to that at follow-up, for a sub-cohort of 12,292 people who completed further data collection between 30 ^th^ April 2014 and 7 ^th^ March 2019. They found high agreement between the job at baseline and at follow-up: 67% for ‘other essential workers’, and 92% for ‘non-essential workers’. For more narrowly defined occupational groups, agreement ranged from 53% for food workers to 88% for healthcare professionals.

*Cohort analyses*

One possible set of analyses for this data is to undertake standard cohort (Poisson regression of Cox regression) analyses with a positive SARS-CoV-2 test as the outcome. Such analyses have been performed by Chadeau-Hyam *et al.* ^35^ who also compared the risk factors for positive COVID-19 tests with those for negative COVID-19 tests (this is discussed further below). Mutambudzi *et al.* ^47^ have performed similar analyses with severe COVID-19 (a +ve test in a hospital setting and/or COVID-19-related death) as the outcome. Thus, they have already published findings for the standard SOC occupational groups but have not published any findings for COVID-19-related JEM.

*Nested case-control (test-negative design)*

An alternative approach to analysing the UK Biobank data would be to use the test-negative design. The rationale for this is that during the first wave of the pandemic testing was done on the basis of symptoms and/or high-risk occupations (e.g. healthcare workers), so standard cohort analyses may be biased (e.g. Chadeau-Hyam *et al.* ^43^ found particularly high positivity rates for healthcare workers which may just reflect that this group was being tested regularly). Chadeau-Hyam *et al.* in part addressed this selection bias by comparing the findings for positive and negative COVID-19 tests (they compare the findings for tested vs non-tested, +ve vs non-tested, -ve vs non-tested, and +ve vs -ve), but such an analysis has not been done for occupation.

### Understanding Society

***Study type: cohort, nested case-control***

*Possible analyses: Poisson regression, Cox proportional hazards model, logistic regression*

Understanding Society is a UK-wide long-term longitudinal study involving approximately 10,000 participants per decade. Understanding Society uses probability sampling and is constructed to allow population inferences. From April 2020, participants from the main Understanding Society sample completed an online survey relating to the COVID-19 pandemic once a month from April to July, and then once every 2 months from September onwards. Each survey includes core content (e.g. SARS-CoV-2 test results and symptoms, information about working from home or furlough) which is designed to track changes. The survey also includes variable content adapted each month as the coronavirus situation develops. The latest release of data was for the September 2020 questionnaire, and at that point 19,763 participants had completed at least one survey. Occupation data was collected in June 2020 and this included 3-digit SOC codes and sector data. The dataset contains information on age, gender, and ethnicity, as well as geographical information. Nandi and Platt ^48^ found that within the Understanding Society population, Black Africans are more likely to report experiencing SARS-CoV-2 symptoms than White UK, and this could not be explained by greater exposure to overcrowding or by the fact that they were keyworkers.

The Understanding Society suite of data sets includes weighting (if necessary) to allow valid population inferences. This includes weighting related to the design (clustering and stratification) and to the response. Weighted analyses may be conducted using SVYDESIGN commands in R.

*Cohort analyses*

One possible set of analyses for this data is to undertake standard cohort (Poisson regression or Cox regression) analyses with either positive SARS-CoV-2 test and/or symptoms suggestive of SARS-CoV-2 as the outcome, and using the 1-digit SOC codes or sector as covariates. Note that this dataset is unlikely to be large enough to consider breakdown by 2-digit SOC codes. Covariates that take into account periods of working from home or furlough can be included (these could be time-varying). Analysis using covariates derived from the JEM can be also included. Symptom data is likely to overestimate the incidence of SARS-CoV-2, however access to testing and motivation to take a test is likely to vary by occupation whereas reporting of symptoms is likely to be independent of occupation.

*Nested case-control (test-negative design)*

An alternative approach to analysing the UK Understanding Society data would be to use the test-negative design. The rationale for this is that during the first wave of the pandemic testing was done on the basis of symptoms and/or high-risk occupations (e.g. healthcare workers), so standard cohort analyses may be biased. Usually once someone has tested positive, they would not be re-tested, and if they were, they would be excluded from the analysis. Thus, the analysis would include all tests of people who had not previously tested positive, and the test+ves and the test-ves would then be compared. Of course, someone may test negative on one date (for which they would be a test-ve control) and test +ve on a subsequent date (for which they would be a test+ve case), but this is allowable under the test-negative design (and density-matched case-control studies in general ^49^), provided that the data are adjusted for date of test.

### OpenSafely

***Study type: cohort, nested case-control***

*Possible analyses: Poisson regression, Cox proportional hazards model, logistic regression*

OpenSafely is a database involving national (England) primary care electronic health record data and is linked to ONS death data. The database includes 17,289,392 adults (male and female who are 18 years and above) currently registered as active participants in a TPP (a healthcare technology company) general practice in England on 1 ^st^ February 2020, and with at least one year of prior follow-up in the GP practice to ensure that baseline characteristics have been adequately captured. The database includes information on age, sex, Body Mass Index (BMI), smoking, and a large number of comorbidities.

Williamson *et al.* ^50^ have analysed the OpenSafely data and linked the primary care records to 10,926 COVID-19-related deaths. They found higher death rates to be related to male sex, older age, higher deprivation, diabetes, severe asthma, and various other medical conditions. Black and South Asian people were at higher risk of COVID-19-related death, even after adjustment for potential confounders.

The ethnic differences were explored further by Mathur *et al.* ^26^ who found substantial evidence of ethnic inequalities in the risk of testing +ve, ICU admission, and mortality, which persisted after accounting for explanatory factors including household size. However, they noted that some of this excess risk may be related to factors not captured in clinical records such as occupation. They note that prioritizing linkage between health, social care and employment data and engaging with ethnic minority communities is essential for generating evidence to prevent further widening of ethnic inequalities in COVID-19.

Thus, OpenSafely is a potentially important database for examining occupational differences in COVID-19 incidence, severity, and mortality, adjusted for other factors such as deprivation and ethnicity. However, occupational information has not been linked to OpenSafely at this stage.

## Conclusions

A large number of data sets are available to potentially assess occupational risks of COVID-19 incidence, severity, or mortality. All have various strengths and weaknesses. For example, mortality data suffer from problems of coding of COVID-19 deaths, and the unavailability (in England and Wales) of deaths that have been referred to the Coroner, and testing data is heavily biased in some periods (particularly the first wave) because some occupations (e.g. healthcare workers) were tested more often than the general population. In principle, random population surveys are ideal for estimating population prevalence and incidence but are also affected by non-response. Thus, any analysis of the risks in a particular occupation or sector (e.g. transport), will require a careful analysis and triangulation of findings across the various available data sets.

## Data Availability

All data underlying the results are available as part of the article and no additional source data are required. OSF: PROTECT initiative extended Covid-19 occupational questionnaire. https://doi.org/10.17605/osf.io/hdc8s ^19^. Data are available under the terms of the Creative Commons Attribution 4.0 International license (CC-BY 4.0).
