# Supplementary material for: Occupational differences in COVID-19 incidence, severity, and mortality in the United Kingdom: Available data and framework for analyses
Source: Wellcome Open Res. 2023 Jan 13;6:102. Originally published 2021 May 10. [Version 2] doi: 10.12688/wellcomeopenres.16729.2 (PMC8188261; doi:10.12688/wellcomeopenres.16729.2)
Supplement: Peer review history

# Peer review: Reviewer response for version 2

**DOI:** 10.21956/wellcomeopenres.20887.r54095

**Published:** 2023-01-27

**Authors:** David Kriebel

**Affiliations:** 1 Department of Public Health, University of Massachusetts Lowell, Lowell, MA, USA

I have reviewed the recent revisions in this article. I would like to amend my status from 'Approved with Reservations' to 'Approved'.

Is the rationale for developing the new method (or application) clearly explained?

Yes

Is the description of the method technically sound?

Partly

Are the conclusions about the method and its performance adequately supported by the findings presented in the article?

Yes

If any results are presented, are all the source data underlying the results available to ensure full reproducibility?

No source data required

Are sufficient details provided to allow replication of the method development and its use by others?

Yes

Reviewer Expertise:

NA

I confirm that I have read this submission and believe that I have an appropriate level of expertise to confirm that it is of an acceptable scientific standard.

# Peer review: Reviewer response for version 1

**DOI:** 10.21956/wellcomeopenres.18450.r43808

**Published:** 2021-06-08

**Authors:** Paul Demers, M. Anne Harris, Jill MacLeod

**Affiliations:** 1 Occupational Cancer Research Centre, Ontario Health, Toronto, Canada; 2 School of Occupational and Public Health, Ryerson University, Toronto, Canada

We read the method article by Neil Pearce and colleagues with great interest, in part because we are moving forward with similar work here in Canada. The paper is a great contribution. Although in many ways it is UK-specific, the broader issues it addresses are relevant to non-UK researchers trying to develop the best methods for approaching this difficult topic. This paper was very useful in organizing our thoughts on the methodological and challenging issues, though we do have some suggestions.

 On page 5, perhaps testing should be added to the list of outcomes to be examined. Although it is not a disease, it is an important indicator for the potential to recognize the disease and testing and test-positivity rates are useful for understanding COVID-19 and the development of public policy.

 OSCAR is a very positive development for future coding of occupations and we look forward to learning more. On the other hand, the automated coding currently used for many large existing data sets can have major problems in terms of both reliability and validity, which increase with the number of digits used. The effect of the misclassification introduced is not differential in regards to disease status, so likely mutes associations. This deserves mention as a limitation of these datasets and highlight the value of OSCAR.

 We were surprised at the lack of discussion of industry sector. Some characteristics of a workplace can sometimes be better characterized by the industry, such as whether the work is “public facing” or “essential” which impact the potential for infection while operating or whether the work continues during lockdown. For example, someone in a cleaning occupation could have a quite different risk depending on whether they are employed in a hospital, factory, restaurant, or recreational facility.

 Although “Confounders and Effect Modifiers” is a heading, the discussion of effect modification is very limited. In particular, the issue of race/ethnicity is extremely important and deserves consideration as an effect modifier. In our country it has a major impact on where people are employed, testing rates, availability of vaccines, and vaccine hesitancy.

 We were surprised at the discussion of geography limited to political regions. Surely other options are available in the UK? One of the major challenges facing us is differentiating workplace from community transmission and geography, at the very least urban versus rural, is a useful surrogate.

 Triangulation is discussed in broad terms. Perhaps an example would be helpful, such as using the population health approaches discussed in the paper with the workplace level information provided by the Public Health England outbreak investigations. 

 Effect modification is not raised in the context of analysis. I assume that the investigators would look at this, but it is important to mention understanding the complex relationship between the variables before treating them as confounders and adjusting away their effects. Again race/ethnicity is an important example but, given differences in testing, vaccination, and other factors, even sex and age deserve close examination before adjusting away their effects. For example, are certain occupational groups infected at an earlier age?

 Although selection bias is mentioned in relation to the UK Biobank, no further discussion of the point is provided, other than it may diminish over time. A major challenge with many similar cohorts is that they are based on voluntary participation and may not be representative of the labour force.

**Minor comments**

 In the first sentence “and the United Kingdom is currently experiencing particularly high infection and death rates.” – suggest change to “has experienced” to not be rooted in one time.

 In Table 1 please specify “UK” in the title. Is the availability of occupational data in REACT still “unknown?” Perhaps “Possible” in the last column could be described more?

 The link for the occupational questionnaire (reference 19) seems to have a description of the questionnaire, but not the questionnaire itself, which would be helpful.

Is the rationale for developing the new method (or application) clearly explained?

Yes

Is the description of the method technically sound?

Yes

Are the conclusions about the method and its performance adequately supported by the findings presented in the article?

Yes

If any results are presented, are all the source data underlying the results available to ensure full reproducibility?

No source data required

Are sufficient details provided to allow replication of the method development and its use by others?

Yes

Reviewer Expertise:

NA

We confirm that we have read this submission and believe that we have an appropriate level of expertise to confirm that it is of an acceptable scientific standard, however we have significant reservations, as outlined above.

# Peer review: Reviewer response for version 1

**DOI:** 10.21956/wellcomeopenres.18450.r43846

**Published:** 2021-05-28

**Authors:** Anthony D. LaMontagne

**Affiliations:** 1 Institute for Health Transformation, School of Health & Social Development, Deakin University, Geelong, Australia

**Summary:**

 This article makes a valuable contribution in detailing a wide range of population-level data sources for investigating occupational differences in COVID-19 incidence, severity, and mortality in the UK.

**Some minor suggestions:**

 Page 3, first paragraph of Introduction:

 “the United Kingdom is currently (as of MONTH, year) experiencing particularly high infection and death

 rates.” Suggest inserting month and year, as situation constantly changing

 Page 5, Methods

 Under ‘Occupational Codes’ section: SOC is presumably specific to the UK? Simply mention that qualification for non-UK international readers, and perhaps note its compatibility/translatability to ISCO (International Standard Classification of Occupations)? 

 Page 6, Table 2:

 Excellent example comparing mortality rates by different types of vehicle drivers, but would be good to include time frame of data (calendar year/months) presented in Table 2 to make clear that this was during COVID pandemic.

 Page 5:

 The OSCAR tool a great innovation—hadn’t heard of this before. Could greatly facilitate systematic collection of occupation data.

 Tried to get a look at the Questionnaire at the “Extended Data” link, but didn’t manage to see the actual questionnaire.

 The JEM development is very promising. ‘Risk factors for transmission’ in the JEM could perhaps also include interaction with members of the public. This would be the case, for example, for workers stacking supermarket shelves. Or distinguish between or indoor [e.g., building] or enclosed space [e.g., public transport bus] proximity with members of the public versus outdoor (e.g., traffic control worker at an inner city construction site)? Such interaction/interfacing should probably be independent of distance, acknowledging the potential for aerosol transmission. Is this what the authors are trying to get at by “c. Indirect contact”? Not clear.

 It’s a finer/minor point, but job insecurity might be better expressed as ‘employment precarity’ because some higher status jobs have low security but relatively good working conditions, whereas precarious employment (such as zero hours contracts) has both low security and a raft of other poor working conditions that could predispose to COVID exposure and infection. Perhaps the focus on zero hours contracts is because there is a source of data on this in the UK by occupation?

 The focus on occupation is well-founded and based on the availability of data as well as historical precedent. But perhaps the authors could consider (if they haven’t already) whether industrial sector information could also be useful, where it is accessible? This could provide another lens on key constructs/risk factors such as precariousness/job insecurity from which to triangulate. For example, the hospitality and retail sectors (in many countries, though not certain about the UK) have a particularly high prevalence of precariously employed workers. CASCOT appears to be able to code sector as well as occupation?

 This article makes a valuable contribution in detailing a wide range of population-level data sources. In seeking to generate relevant measures from these various sources, a possibly useful distinction could be identifying those measures of infection/morbidity/mortality occurrence that are based on the same occupation ‘measurement method’ for numerators and denominators, or cases and non-cases in the populations from which cases have emerged (such rates by occupation based on APS data with comparably SOC-coded occupation for cases and non-cases). These can still be biased, but would at least be internally consistent in exposure (occupation) measurement. We face the same challenges in estimating suicide rates among workers in particular occupations or sectors (e.g., building and construction) based on Coronial investigation records to determine the occupation of suicide cases, while sourcing occupation or sector denominator data from periodic (~every 3-5 years) Labour Force and Census surveys, leaving all sorts of room for error. 

**Correction?**

 Please check the links. AT least one needs to be more specific: the hyperlink from OSCAR (Occupational Self-Coding and Automatic Recoding) took me to a web page for “Lungs at Work”, not a description or report on OSCAR (whereas the CASCOT link does go to a CASCOT-specific page).

Is the rationale for developing the new method (or application) clearly explained?

Yes

Is the description of the method technically sound?

Yes

Are the conclusions about the method and its performance adequately supported by the findings presented in the article?

Yes

If any results are presented, are all the source data underlying the results available to ensure full reproducibility?

Yes

Are sufficient details provided to allow replication of the method development and its use by others?

Yes

Reviewer Expertise:

NA

I confirm that I have read this submission and believe that I have an appropriate level of expertise to confirm that it is of an acceptable scientific standard.

# Peer review: Reviewer response for version 1

**DOI:** 10.21956/wellcomeopenres.18450.r43812

**Published:** 2021-05-25

**Authors:** David Kriebel

**Affiliations:** 1 Department of Public Health, University of Massachusetts Lowell, Lowell, MA, USA

This is a very useful summary of a large number of resources available in the U.K. for studies of occupational differences in Covid-19. The topic is highly relevant because the roles of occupation in risk of Covid-19 are complex, and unfortunately these roles have not been sufficiently taken into consideration in public debates and policy formulation. The authors are very qualified to provide a thorough overview of the topic with a valuable compendium of resources both in data and in methods.

 One substantive addition to the paper would strengthen it significantly. The discussion of exposure variables could be strengthened. The paper lacks reference to the literature on different ways of assessing exposure to SARS-CoV-2 through occupational characteristics like interacting with the public, working on a production line in close proximity to other workers, and by being in a so-called "essential" occupation. Some examples of exposure assessments that would strengthen the paper include ^1^^-^^5^.

 A few minor additional suggestions:

- In the second paragraph, it would be helpful to state explicitly that the data resources are for the U.K.
- In the discussion of race/ethnicity on page 6, and/or in the discussion of confounders/effect modifiers on page 8, I think that it would be helpful to go into more detail about the complex potential roles of race/ethnicity (and I suppose also deprivation) in the pandemic. It is not at all a simple matter to "control" for race/ethnicity when it may affect risk of infection, underlying conditions, probability of being tested, quality of health care, and probably several other critical steps. Hawkins *et al*. ^6^ found that Blacks consistently had higher mortality rates from Covid-19 than Whites within the same occupation, in Massachusetts USA. There are several possible reasons for this, but I think the paper would be improved by acknowledging the complexity of teasing out the reasons for race/ethnic differences. 
- On page 5, the application of wastewater epidemiology to workplaces is a good point to raise, and I think there might be a few additional references that could point readers to concrete examples. Prisons and other congregate settings are being studied effectively to identify outbreaks, and these of course are occupational exposures.

Is the rationale for developing the new method (or application) clearly explained?

Yes

Is the description of the method technically sound?

Partly

Are the conclusions about the method and its performance adequately supported by the findings presented in the article?

Yes

If any results are presented, are all the source data underlying the results available to ensure full reproducibility?

No source data required

Are sufficient details provided to allow replication of the method development and its use by others?

Yes

Reviewer Expertise:

Occupational and environmental exposure assessment and epidemiology.

I confirm that I have read this submission and believe that I have an appropriate level of expertise to confirm that it is of an acceptable scientific standard, however I have significant reservations, as outlined above.

## References

[1] : Nonrelocatable Occupations at Increased Risk During Pandemics: United States, 2018. *Am J Public Health* .110(8) : 10.2105/AJPH.2020.305738 1126-1132 10.2105/AJPH.2020.305738 32552016PMC7349441

[2] : Estimating the burden of United States workers exposed to infection or disease: A key factor in containing risk of COVID-19 infection. *PLoS One* .2020;15(4) : 10.1371/journal.pone.0232452 e0232452 10.1371/journal.pone.0232452 32343747PMC7188235

[3] : Prevalence of Underlying Medical Conditions Among Selected Essential Critical Infrastructure Workers - Behavioral Risk Factor Surveillance System, 31 States, 2017-2018. *MMWR Morb Mortal Wkly Rep* .2020;69(36) : 10.15585/mmwr.mm6936a3 1244-1249 10.15585/mmwr.mm6936a3 32914769PMC7499835

[4] : Occupations by Proximity and Indoor/Outdoor Work: Relevance to COVID-19 in All Workers and Black/Hispanic Workers. *Am J Prev Med* .60(5) : 10.1016/j.amepre.2020.12.016 621-628 10.1016/j.amepre.2020.12.016 33745817PMC7970652

[5] : Social Determinants of COVID-19 in Massachusetts, United States: An Ecological Study. *J Prev Med Public Health* .2020;53(4) : 10.3961/jpmph.20.256 220-227 10.3961/jpmph.20.256 32752590PMC7411251

[6] : COVID-19 deaths by occupation, Massachusetts, March 1-July 31, 2020. *Am J Ind Med* .64(4) : 10.1002/ajim.23227 238-244 10.1002/ajim.23227 33522627PMC8013546
